# Lidocaine inhibits osteoclast activity in vitro

**DOI:** 10.1016/j.bonr.2026.101935

**Published:** 2026-07-11

**Authors:** Stan Tuijp, Marc Meijer, Ineke D.C. Jansen

**Affiliations:** a Department of Periodontology, Academic Center for Dentistry Amsterdam (ACTA), University of Amsterdam and VU University, Amsterdam, the Netherlands

**Keywords:** Osteoclasts, Lidocaine, Inflammation, Gingiva fibroblasts

## Abstract

Local anesthetics such as lidocaine have anti-inflammatory effects. However, the anti-inflammatory mechanism remains vague, but phagocytosis, migration, exocytosis, cellular metabolism and cellular pH levels seem to be affected. During COVID-19 pandemic was found that pro-inflammatory serum levels of IL-1, IL-6, TNF-α were reduced when lidocaine was given to these patients. These pro-inflammatory markers are also elevated in periodontitis and osteoporosis. IL-1 is known as an osteoclast activating factor. To investigate if lidocaine can inhibit osteoclast activity lidocaine was added to osteoclast precursors (PBMCs) alone or in co-cultures with GF cells. Lidocaine was added in a concentration of 3 mM for 6 h and 6 mM for 24 h. After 3 weeks of culture osteoclast formation, gene expression of RANKL, and inflammatory cytokines and resorption activity were measured.

Exposure for 24 h to 6 mM lidocaine showed large vacuoles in both cell types. After three weeks of culture osteoclasts were formed but their number was significantly lower in the with 6 mM lidocaine treated co-cultures. Also, gene expression of RANKL, MCP-1, IL-1 and TNF-α was lower. When a 3 mM lidocaine concentration was added for 6 h the vacuoles were only visible in the GF cells and not in the PBMCs and after 3 weeks the number of osteoclasts formed was not reduced. When osteoclasts cultured on bone their resorptive activity was significantly lower.

The lower lidocaine concentration and shorter incubation time showed that cell number was not affected but resorption activity of the osteoclasts was decreased. This indicates that lidocaine can inhibit osteoclast activity.

## 1 Introduction

Periodontitis and osteoporosis have in common that they are both age related, inflammation driven diseases characterized by increased bone degradation (Yu and Wang, 2022; Lerner, 2006).

Healthy bone is continuously remodeled in which bone resorption, by osteoclasts, is in balance with bone formation, by osteoblasts, a process being referred as coupling (Parfitt, 1982). Under inflammatory conditions bone resorption is stimulated and bone formation inhibited thus an imbalance of bone turnover resulting a net loss of bone (Parfitt, 1982). This enhanced bone resorption is caused by pro-inflammatory cytokines such as IL-1 and TNF-α which stimulates bone resorption and inhibits bone formation, resulting in net bone loss (Lerner, 2006; Bertolini et al., 1986; Nguyen et al., 1991; Zhang et al., 2020; Zupan et al., 2013).

The local anaesthetic lidocaine is found to be a potent anti-inflammatory drug comparable with steroids and non-steroidal anti-inflammatory drugs (NSAIDS) (Wickström et al., 2017). The anti-inflammatory mechanism remains vague; however, phagocytosis, migration, exocytosis and cellular metabolism seem to be affected. Also pH dysregulation is suggested (Ali and El-Mallakh, 2020; Caracas et al., 2009; Karnina et al., 2021; Peck et al., 1985).

Local anesthetics, such as lidocaine, is widely used in dentistry and in other medical disciplines. They block neuronal action potentials, which prevent the proper transmission of signals by the nerves, thereby decreasing pain signaling to the central nervous system. During the COVID-19-pandemic lidocaine was used as an anti-inflammatory agent suppressing cough (Aminnejad et al., 2020). COVID-19 is characterized by a cytokine storm, in which several interleukins (i.e. IL-1, IL-6) and tumor necrosis factor-α (TNFα) are elevated (Hu et al., 2021). Also, in osteoporosis and periodontitis these cytokines are elevated (Zupan et al., 2013; De Martinis et al., 2020; Xu et al., 2023).

Whether lidocaine also restores the imbalance between bone resorption and formation resulting from inflammation is currently unknown.

To investigate if lidocaine can inhibit excessive bone degradation in above mentioned inflammatory diseases gingiva fibroblasts were used to mimic osteoblasts, since these gingiva fibroblasts also express, just like osteoblasts and osteocytes, RANKL and OPG, factors important for communication with osteoclast-precursors to trigger osteoclastogenesis (Wielento et al., 2023). To investigate the effect of lidocaine on bone turnover GF cells were co-cultured with PBMCs as osteoclast-precursors. GF cells express a variety of molecules known to be involved in osteoclastogenesis, including macrophage colony-stimulating factor (M-CSF) and receptor activator of nuclear factor kappa-B ligand (RANKL) (Sjöström et al., 2000).

The aim of the current study is to determine whether lidocaine restore the imbalance in bone formation and degradation resulting from inflammation. If successful, then patients suffering from inflammatory disease and/or bone degradation would greatly benefit from lidocaine treatment.

## 2 Materials and methods

### 2.1 Gingival fibroblasts

GF were obtained from four healthy donors (males and females with an age between 17 and 45 years) who underwent extraction of a third molar. The study was conducted in accordance with the Declaration of Helsinki and the protocol was approved by the Ethical Review Board of the VU Medical Center, Amsterdam (now Amsterdam UMC), the Netherlands (number 2016/105). Signed informed consent was obtained from all individuals. Periodontal tissues around the molars showed no overt signs of gingival inflammation or periodontitis (no plaque, periodontal probing ≤3 mm, no bleeding on probing, and no sign of loss of attachment).

Free gingiva and part of the intradental gingiva was cut off the tooth by means of a scalpel-knife and chopped into fragments of approximately 1 mm. The tissue fragments were washed twice in DMEM (DMEM, Gibco BRL, Paisley, Scotland) supplemented with 10% FC1 (HyClone, Logan, UT), and 1% antibiotics (100 U/mL penicillin, 100 mg/mL streptomycin, and 250 ng/mL amphotericin B) (Antibiotic antimycotic solution, Sigma, St. Louis, MO). The biopsies were cut into small pieces and divided in a 6-well dish with 1.5 mL DMEM +10% FCS +1% antibiotics. The 6-well dishes were placed in a humidified atmosphere of 5% CO2 in air at 37 °C. GF were expanded for 3 passages and aliquots were stored in liquid nitrogen. In all experiments were performed with GF cells between the 4-7th passage.

### 2.2 PBMC isolation

Peripheral blood mononuclear cells (PBMCs) were isolated from buffy coats form healthy donors (Sanquin, Amsterdam, The Netherlands) (ethical committee number NVT230.01). The buffy coat was diluted 1:1 in PBS (Gibco) containing 1% citrate buffer (Merck, Darmstadt, Germany). Twenty-five mL of diluted blood was carefully layered on 15 mL lymphoprep (Axisshield Po CAS, Oslo, Norway) and centrifuged for 30 min at 1200*g* without brake. The interphase containing PBMCs was washed two times in PBS + 1% citrate buffer and finally recovered in αMEM (Gibco) + 10% FC1 (Hyclone) + 1% antibiotics (Sigma-Aldrich). The cells that were not used were frozen in 10% DMSO (Sigma) and 90% FC1 (Hyclone) o/n in a Mr. Frosty cryo-container to freeze the samples slowly at 1 degree of cooling per minute (Nalgene by Thermo Fisher Waltham, MA) stored at −80 °C for one day and subsequently stored in liquid nitrogen. Freshly isolated PBMCs or defrosted PBMCs were used in the experiments.

### 2.3 (Co-)culture experiments

All experiments were performed in 48 or 96 well plates (Greiner bio-one),

The GF cells were seeded at a density of 3 × 10^4^ cells/well and kept overnight to attach at 37 °C in a humidified atmosphere with 5% CO_2_ in αMEM without phenol red (Gibco) + 10% FC1 (Hyclone) + 1% antibiotics (Sigma) before PBMCs were added.

The next day PBMCs were plated on top of the GF cells or in a separate well at a concentration of 1 × 10^6^ cells/well and in 37 °C in a humidified atmosphere with 5% CO_2_. αMEM without phenol red (Gibco) + 10% FC1 (Hyclone) + 1% antibiotics (Sigma) supplemented with 25 ng/mL human recombinant M-CSF (R&D systems, Minneapolis, MN) for the first 3 days.

After 3 days the following cells were cultured in: -1-. **control** medium containing αMEM without phenol red (Gibco) + 10% FC1 (Hyclone) + 1% antibiotics (Sigma); -2-. “**inflammatory**” condition: control medium containing 100 ng/mL LPS from *P. gingivalis* (Sigma-Aldrich) or 10 ng/mL IL-1β (R&D); -3-. **Inflammatory condition plus lignospan/lidocaine**; same as in -2-, but with up to 6 mM lidocaine (Septodont, Brussels, Belgium and Thermofisher). After 6 h or 24 h media were removed and replaced for αMEM without phenol red (Gibco) + 10% FC1 (Hyclone) + 1% antibiotics (Sigma). In the co-cultures 10 nM vitamin D (Sigma-Aldrich) was added to stimulate the GF cells to express RANKL. In the PBMC monocultures 10 ng/mL M-CSF (R&D) and 5 ng/mL RANKL (R&D) was added. The media were refreshed twice a week.

For repeated treatment with lidocaine the cells were incubated one week after the first treatment for a second time with lidocaine as described above.

### 2.4 Lysosome visualization

To visualize lysosomes lysotracker red DND-99 (Thermofisher) was added to the cells in culture according to the company's protocol. In short, 50 nM of lysotracker in prewarmed culture medium was added to the cells and incubated for 30 min in the dark at 37 °C. Then lysotracker medium was removed and replaced for fresh culture medium and the fluorescent staining was visualized with a converted fluorescence microscope with the appropriate filter (IMDR Leica).

### 2.5 TRAcP staining- osteoclast quantification

Osteoclast quantification was performed after 21 days of culturing. Cells were fixed in 4% PBS-buffered formaldehyde and stained for the presence of tartrate-resistant acid phosphatase (TRAcP) using the Acid Phosphatase Leukocyte kit (Sigma-Aldrich), following the instructions of the manufacturer. Nuclei were stained with diamidino-2-phenylindole dihydrochloride (DAPI, Sigma-Aldrich). Micrographs were taken from five fixed positions per well using an inverted microscope (Leica Microsystems, Wetzlar, Germany) equipped with a digital camera (Leica DFC320, Leica Microsystems) and analyzed for the number of TRAcP-positive multinucleated cells. Multinucleated TRAcP+ cells with three or more nuclei were considered osteoclasts. Osteoclasts were categorised in three groups: 3–5 nuclei; 6–10 nuclei and ≥ 10 nuclei per cell. Each condition was performed in duplicate and GF cells in co-cultures and PBMCs in monocultures were from four different donors. The mean of duplicate wells was used for analysis.

### 2.6 RNA analysis and real-time quantitative PCR

Quantitative polymerase chain reaction (qPCR) analysis was performed at day 7 and 21. At these time points the culture medium was removed and 300 μL of RNA lysis buffer (Qiagen, Hilden, Germany) was added per well. Subsequently, the plates were stored in −80 °C until further use. RNA isolation was performed using the RNeasy Mini Kit (Qiagen) according to the manufacturer's instructions. The RNA concentration and quality were determined using absorption measurements at 260 and 280 nm with Synergy HT spectrophotometer (BioTek Instruments Inc., Winooski, VT). RNA was reverse transcribed to cDNA using the MBI Fermentas cDNA Synthesis Kit (Vilnius, Lithuania) following the manufacturer's instructions, using both the Oligo(dT)18 and D(N)6 primers.

Real-time PCR was performed on a LC480 Light Cycler (Roche, Basel, Switzerland). Hypoxanthine phosphoribosyltransferase (HPRT) was used as a housekeeping gene. Expression of this gene was not affected by the experimental conditions. The primer sequences used for analysis are listed in Table 1. All PCR efficiencies were comparable. Expression of the genes was normalized for PBGD expression following the comparative threshold (Ct) method. ΔCt (C_t gene of interest_ - C_t PBGD_) was calculated and relative expression of the genes was determined as 2^−(ΔCt)^.

**Table 1:** Primer sequences used for quantitative polymerase chain reaction (qPCR).

Gene	Sequence 5′-3′	Amplicon length (bp)	Ensemble Gene ID
*HPRT*	F:GCTGACCTGCTGGATTACAT R: CTTGCGACCTTGACCATCT	260	ENSG00000165704
*RANKL*	F: CATCCCATCTggTTCCCATAA R: gCCCAACCCCGATCATg	60	ENSG00000120659
*MCP-1*	F: CAgCCAgATgCAATCAATgC R: TgCTgCTggTgATTCTTCTATAgCT	102	ENSG00000108691
*IL-1*	F:TggAgCAACAAgTggTgTTCT R:gAgAggTgCTgATgTACCAgTT	270	ENSG00000125538
*TNF-α*	F: CCCAgggACCTCTCTCTAATCA R: gCTTgAgggTTTgCTACAACATg	103	ENSG00000111956

*HPRT*, hypoxanthine phosphoribosyltransferase; *IL-1 beta*, interleukine 1β; *RANKL*; *MCP-1*, monocyte chemoattractant protein-1; *TNF-α*, tumor necrosis factor α.

### 2.7 Bone resorption

Resorption was measured on slices of bovine cortical bone 650 μm thick and fit into a 96-well plate. PBMCs were cultured on these bone slices for 21 days with M-CSF and RANKL and without/with IL-1 and Lidocaine, as mentioned above. After this period, the cells were removed with 0.25 M NH_4_OH. The slices were washed in distilled water, incubated in a saturated alum (KAl(SO4)2•12H2O) solution, washed in distilled water, and stained with Coomassie Brilliant Blue. Resorption pits were visualized by light microscopy (Leica DFC320). Micrographs of the resorbed area were made with SI-17 × 10 magnification. The total resorbed area was quantified using Adobe photoshop (version 2025Adobe.com) and are presented as a percentage of the total area.

### 2.8 Statistical analysis

All data were analyzed using GraphPad Prism software version 10 (Graphpad Software, San Diego, CA, USA). One way ANOVA with a Tukey's multiple comparisons test was used to compare the 3 groups. Values were considered to be significantly different when *p* ≤ 0.05.

## 3 Results

### 3.1 Long/high exposure to lidocaine modulates cell morphology

**Fig. 1:**
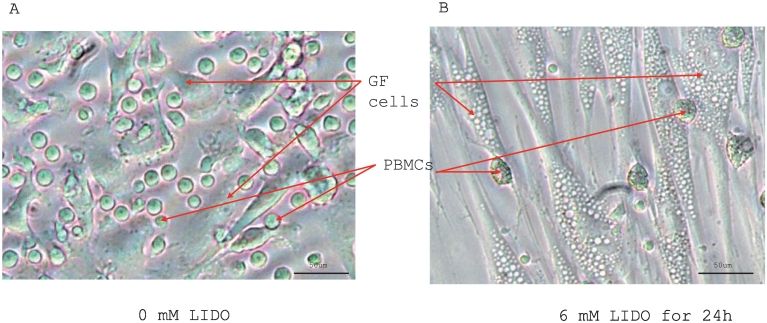
Visualization of a co-culture of PBMCs and gingiva fibroblasts incubated for 24 h with 0 or 6 mM lidocaine. Both cell types, gingiva fibroblasts and PBMCs showed enlarged vacuoles just after treatment with 6 mM lidocaine for 24 h. A. control without lidocaine B. cells with 6 mM lidocaine for 6 h.

**Fig. 2:**
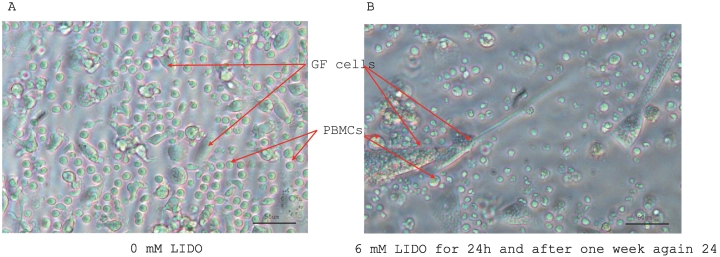
Visualization of a co-culture of PBMCs and gingiva fibroblasts after repeated treatment with lidocaine. A. without lidocaine no enlarged vesicles in GF and PBMCs were present. B. When lidocaine was added again, after a week to the co-culture, less GF and PBMCs were present and the cells were enlarged and occupied with large vacuoles.

**Fig. 3:**
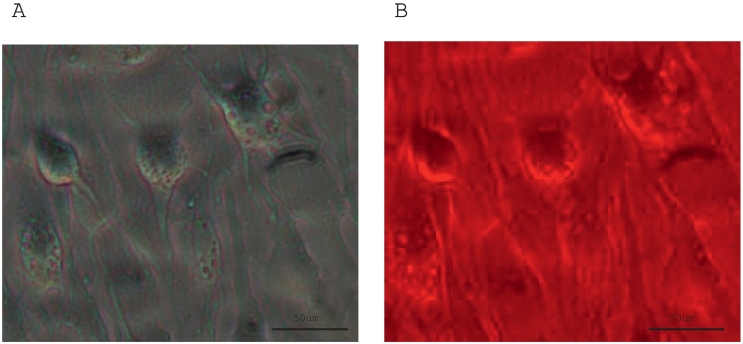
Visualization with lysotracker-red of the lysosomes when cells were treated with lidocaine. | A. Enlarged vesicles in the culture with 6 mM lidocaine. B. Same area after lysotracker-red staining. Some of the enlarged vesicles were stained with lysotracker-red.

The two cell types in the co-cultures treated with 6 mM lidocaine for 24 h showed a different morphology. In the cells were large vesicles present (Fig. 1). When the cells were treated after a week again with the same concentration and time with lidocaine the vesicles were present, but it is also visible that less cells are present (Fig. 2). To visualize the lysosomes and to investigate if these enlarged vesicles were indeed lysosomes lysotracker was added (Fig. 3). We found that some of the lysosomes were stained with lysotracker but not all.

### 3.2 Lidocaine decreases the gene expression of RANKL and some inflammatory cytokines in a co-culture of PBMCs and gingiva fibroblasts when treated with 6 mM lidocaine for 24 h

**Fig. 4:**
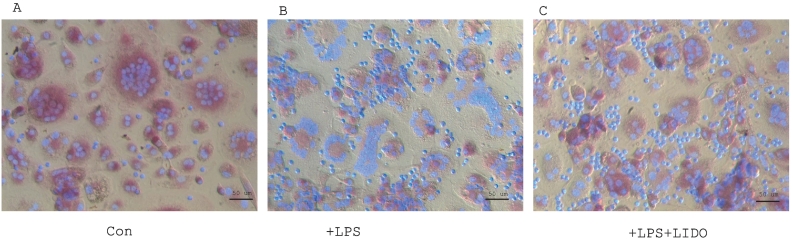
Osteoclasts stained for TRAcP (purple) and with DAPI to visualize nuclei (blue). A = control; B = LPS; C = LPS + lidocaine. Large osteoclasts with many nuclei were present in A and B. In C it seems that the osteoclasts are smaller with less nuclei and more mononuclear cells are present.

**Fig. 5:**
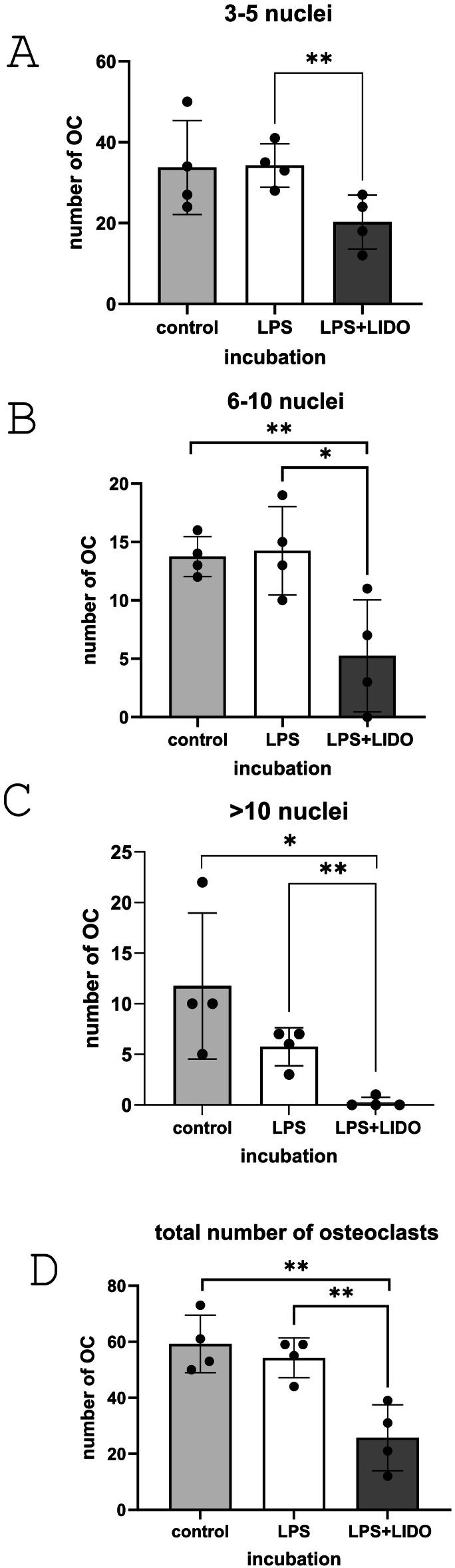
Osteoclast numbers in co-cultures with LPS and lidocaine 6 mM for 24 h. A significant decrease in osteoclast number is seen in the categories 6–10 (B), >10 nuclei (C). Also the total number of osteoclasts (D) was decreased in when lidocaine was added. Only the number of small osteoclasts with 3–5 nuclei (A) was not changed. *p < 0.05; **p < 0.01.

After 3 weeks of culture the cells were stained for TRAcP and the number of osteoclasts formed in the presence or absence of lidocaine was counted. When the PBMCs in a co-culture with gingiva fibroblasts were exposed to 6 mM lidocaine for 24 h and subsequently cultured for 3 weeks with only vitamin D the number of osteoclasts in all categories was significantly lower compared to the cultures with only the inflammatory factor LPS (Fig. 4, Fig. 5). In Fig. 4 TRAcP stained osteoclasts were visible. The number of osteoclasts were counted and divided over 3 categories: osteoclasts with 3–5 nuclei, with 6–10 nuclei and with >10 nuclei and the total number of multinucleated osteoclasts is presented in Fig. 5. In all the categories significantly less osteoclasts were present when cells were cultured with 6 mM lidocaine.

**Fig. 6:**
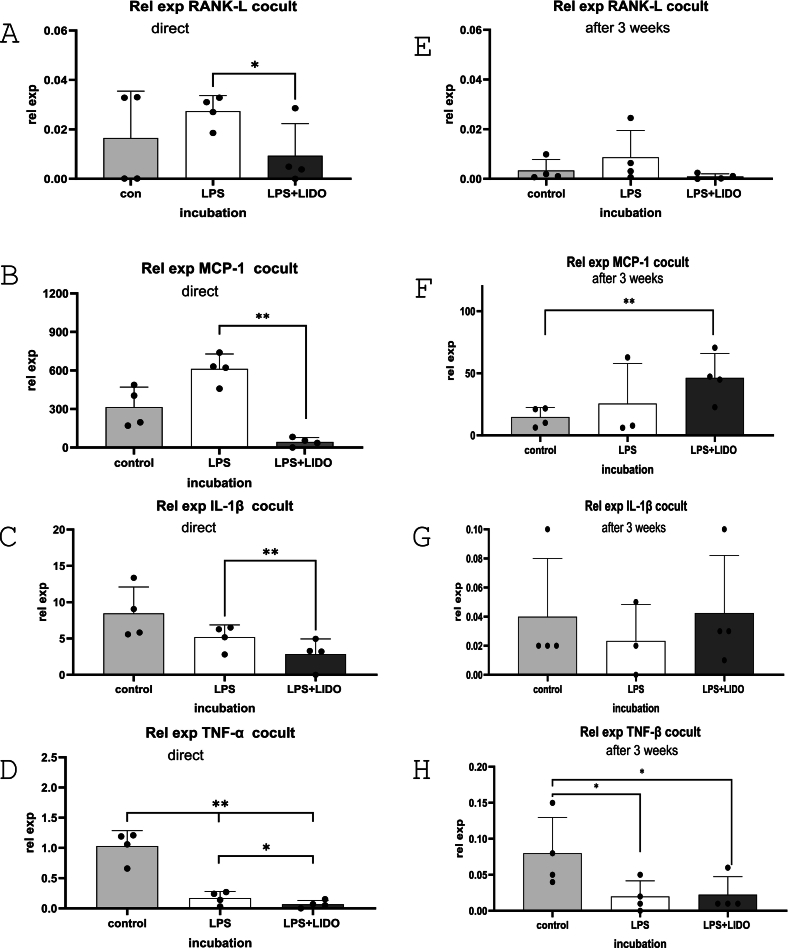
qPCR expression of RANKL, MCP-1, IL-1β and TNF-α in co-cultures with or without 24 h 6 mM lidocaine and LPS direct and after culturing for three more weeks. A-D Expression immediately after lidocaine treatment. A. Relative expression of RANKL. A significant decline of the expression when the co-culture was incubated with lidocaine compared to the culture with only the inflammatory factor LPS. No significant difference was present between control and inflammatory condition. Inflammation induced gene expression of MCP-1 (B), IL-1β (C) and TNF-α (D)were significant lower when lidocaine was present. E–H expression after 3 weeks of culturing. E. Relative expression of RANKL is not different in the lidocaine compared to control and IL-1 treatment. For the inflammatory cytokines MCP-1 (F) is even higher after lidocaine treatment, IL-1β (G) is not changed and only TNF-α (H) is still lower after lidocaine treatment. *p < 0.05; **p < 0.01.

Relative expression was measured in the co-cultures direct after the treatment with lidocaine and after 3 weeks when the cultures were stopped and the number of osteoclasts was counted. qPCR data showed a significant lower RANKL expression compared to LPS treatment just after treatment with lidocaine (Fig. 6A). Also, the inflammatory markers MCP-1, IL-1β and TNF-α were lower at this timepoint compared to LPS treatment. Most of tested genes the expression went down comparable or even lower than the level measured in the control cultures.

**Fig. 7:**
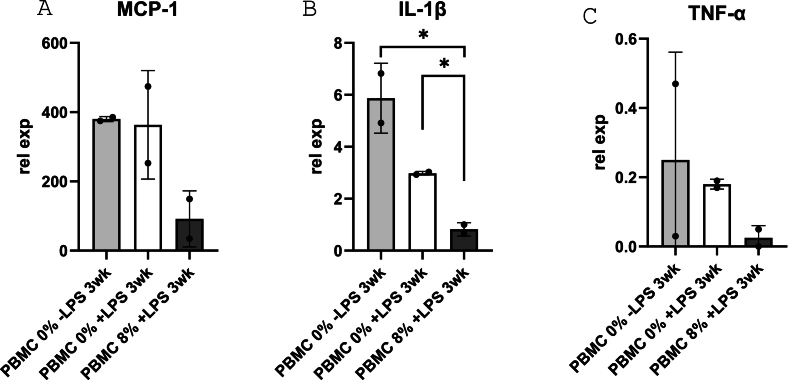
qPCR expression of MCP-1, IL-1β and TNF-α in PBMCs with 24 h 6 mM lidocaine and LPS after culturing for three more weeks. IL-1β and MCP-1 expression were significantly lower after lidocaine treatment *p < 0.05. TNF-α showed a trend of lower expression after lidocaine treatment but not significant.

When the same genes were measured after 3 weeks when the osteoclasts were formed in these co-cultures most of these differences were not present anymore (Fig. 6E–H). MCP-1 expression is even higher compared to the control and LPS incubation (Fig. 6F). Only TNF-α is significantly lower compared to LPS and control (Fig. 6H). However, when the PBMCs were cultured alone and the inflammatory genes were measured after 3 weeks of culture still the same pattern is present as in the co-cultures at timepoint zero (Fig. 7A, B, C). The expression of MCP-1 and IL-1 was significant lower after treatment with lidocaine compared to LPS treatment (Fig. 7A, B). For TNF-α there is only a trend visible, but this might be due to the high standard deviation for the control incubation (Fig. 7C).

### 3.3 Lower concentration and shorter incubation with lidocaine decreases osteoclast activity in co-cultures

**Fig. 8:**
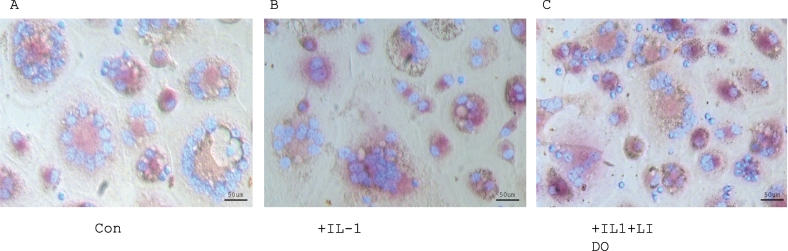
Osteoclast stained for TRAcP and nuclei with DAPI in co-cultures with IL-1β and 3 mM lidocaine for 6 h. A = control; B = IL-1; C = IL1 + lidocaine. Large osteoclasts with many nuclei were present in all the incubations A, B and C.

**Fig. 9:**
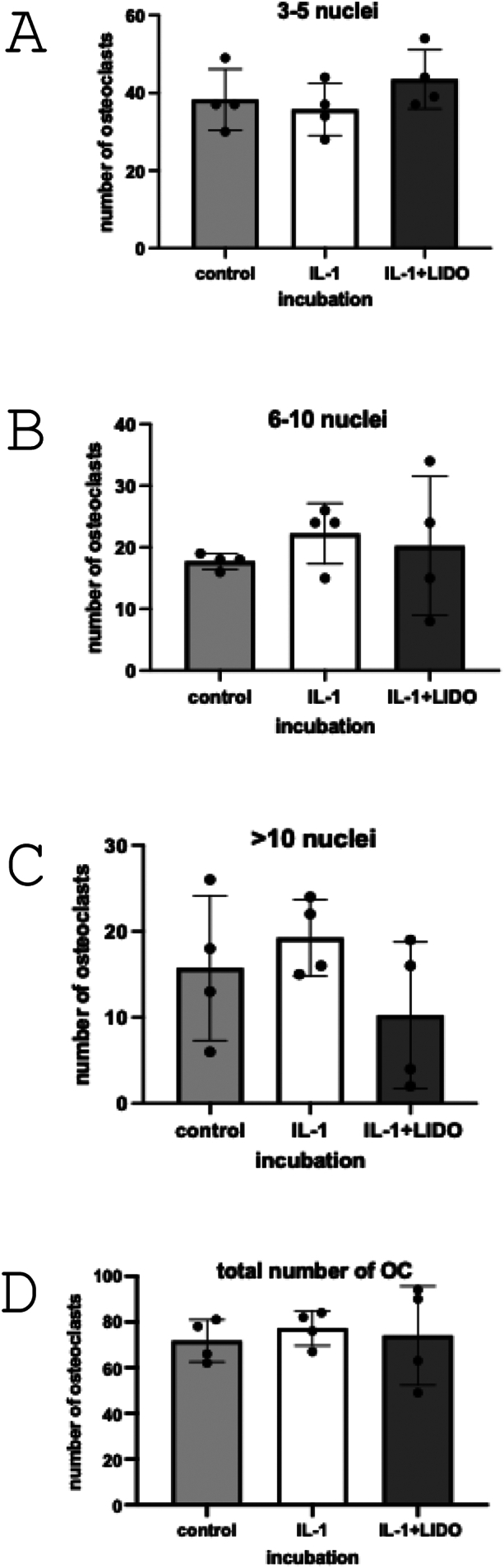
Osteoclast numbers in co-cultures with IL-1β and 3 mM lidocaine for 6 h. No significant differences in osteoclast number is seen in all the categories (3–5 (A), 6–10 (B), >10 nuclei (C) and total number of osteoclasts (D)).

**Fig. 10:**
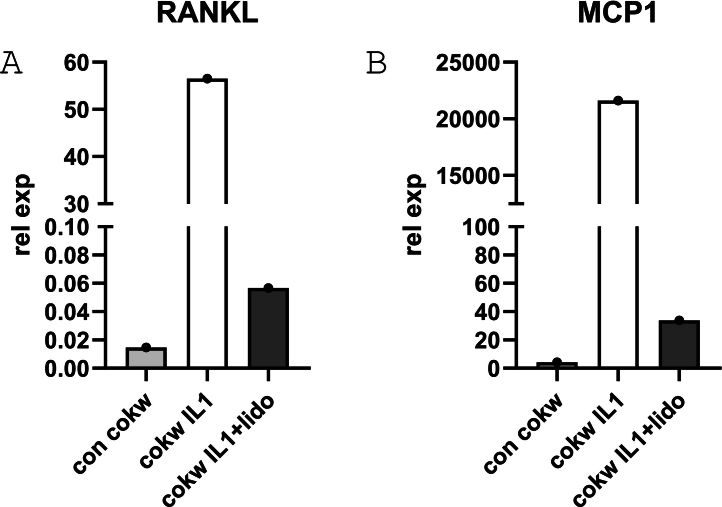
qPCR expression of RANKL and MCP-1, in co-culture with or without 6 h 3 mM lidocaine and IL-1β after culturing for three more weeks. RANKL and MCP-1 expression is nearly back to the control level when lidocaine was added to the with IL-1 treated co-cultures.

Since is known that the cells might be die by high and long lidocaine exposure in the following experiments 3 mM lidocaine was used, and the incubation time was shorter only 6 h. These vesicles visible in the high and long exposure to lidocaine were not visible in PBMCs when incubated with lidocaine concentrations of 3 mM, but still visible in the gingiva fibroblasts. No significant differences were found in the osteoclast numbers for all the categories when the cells were co-cultured with GF cells on plastic. Fig. 8 shows osteoclasts stained for TRAcP. Fig. 9 shows the number of osteoclasts. In all the different categories (3–5, 6–10 > 10 nuclei (A,B,C)) no differences in osteoclast number were found. Also, the total number of osteoclasts (D) was not changed after short incubation with 3 mM lidocaine. The qPCR data showed that RANKL and MCP-1 expression is nearly back to the control level when lidocaine was added to the with IL-1β treated co-cultures (Fig. 10A, B).

### 3.4 Lidocaine inhibits osteoclast resorption activity but not formation when cultured on cortical bone slices

**Fig. 11:**
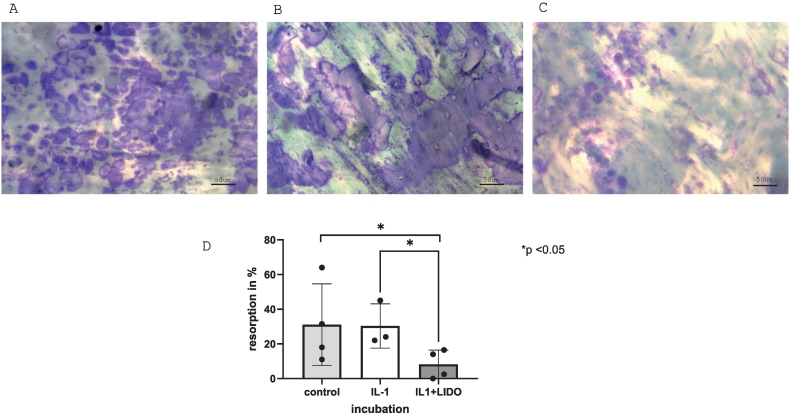
Resorbed area of bone slices by osteoclasts cultured for 21 days without and with 3 mM lidocaine. The resorbed area is stained with Coomassie brilliant blue. A. control, B. IL-1β, C. Il-1β + 3 mM Lidocaine. Less blue spots, indicating resorption pits, are visible on the bone slices incubated with lidocaine. The measured resorption area is plotted in D. The resorptive activity is significantly less by the cells incubated with 3 mM lidocaine for 6 h. *p < 0.05.

When PBMCs alone were cultured on bone slices also no significant differences in osteoclast numbers was found when treated with lidocaine (not shown) but osteoclast activity (resorption) was significant less when the cells were treated with lidocaine (Fig. 11). Fig. 11 shows the resorption pits (blue) made by the osteoclasts after 3 weeks of culturing. The PBMCs were treated with 3 mM lidocaine for 6 h at timepoint zero. After that they cells were cultured for 20 more days on the bone slices. The area of resorption was measured and plotted in Fig. 11D.

## 4 Discussion

When the cells were treated with 6 mM lidocaine for 24 h the lysosomes of both cell types enlarged. Pena et al. (Pena et al., 1982) described this earlier for fibroblasts as “amine trapping”, meaning that lidocaine is entrapped in the lysosomes. Amine trapping is a process in which a.

pH-neutral drug can pass the lysosomal membrane and then becomes protonated in the acidic environment which traps it in its charged form, preventing it from diffusing back out into the cell's cytosol (Kazmi et al., 2013). The accumulation of drugs in lysosomes can lead to prolonged exposure and may lead to cell death (Turk and Turk, 2009; de Duve et al., 1974).

Pena et al. (1982) suggested that exposure of cultured human skin fibroblasts to lidocaine in concentrations over 3 mM for more than 6 h caused dramatic vacuolation of the cells. The vacuoles were formed by expansion of lysosomes. Some of the lysosomes are fluorescently stained with lysotracker red but not all of them. It is known that lysotrackers only accumulate in cellular compartments with low internal pH. Since the pH might be higher in the trapped lysosomes these lysosomes will be less sensitive for lysotracker (Pena et al., 1982). This might be the explanation why not all the enlarged lysosomes are stained with lysotracker.

Cytotoxicity of local anesthetics is described in the review (Zhang et al., 2023). Various cell types, concentrations and exposure times are tested. For lidocaine >1% causes cell death when exposed for more than 1 h (Zhang et al., 2023). The decline of osteoclast formation in our experiments in all the categories found in the high and long exposure of lidocaine might be caused by apoptosis of the PBMCs due to amine trapping. However, the gene expression of the inflammatory factors MCP-1 and IL-1 cannot be explained by apoptosis because relative gene expression is related to the expression of the housekeeping gene HPRT. The expression of HPRT was very consistent and not changed by lidocaine treatment. It may still be possible that lidocaine treatment is lowering the inflammation and subsequently osteoclast activity. However, the formation of enlarged lysosomes is undesirable when treating patients with a dose of lidocaine that causes the afore mentioned side effects; therefore, we decided to repeat the experiments with a lower concentration of lidocaine (3 mM instead of 6 mM) and a shorter incubation time of only 6 h. In these experiments the gingiva fibroblasts still showed enlarged lysosomes, but not the PBMCs.

In the lower exposure experiments the number of osteoclasts formed after 3 weeks of culture were not changed when lidocaine was present compared to the control cultures, when the cells we cultured on plastic. Normal numbers of osteoclasts were formed, indicating that the precursors remained normally viable. The relative expression of the inflammatory cytokines is comparable to the results found at the higher concentration of lidocaine. The expression of RANKL and MCP-1 seems to be lower after lidocaine treatment. This difference was not significant, likely due to the small sample size.

During inflammatory diseases pro-inflammatory cytokines such as IL-1, TNF are increased and causing an increase of RANKL leading to enhanced bone resorption (Tuladhar et al., 2025; Boyle et al., 2003).

When the PBMCs were cultured alone on bone without and with lidocaine, the number of osteoclasts formed are comparable in all the treatments. However, the resorptive activity of the formed osteoclasts was significantly less for the cells treated with lidocaine.

Osteoclasts resorb the bone by making a ruffled border area surrounded by a sealing zone in which lysosomal enzymes are secreted (Everts et al., 2022). These enzymes dissolve the mineral in the bone. It is possible that due to lidocaine the acidification of the ruffled border area is not properly working and therefore resorption is inhibited. In pycnodysostosis patients, where cathepsin K is not present, or when cathepsin K is inhibited only shallow resorption pits are formed and not what is normally seen resorption trenches (Mons et al., 2019). With lidocaine we also found shallow resorption pits, which may indicate that indeed lysosomal dysfunction causes the decreased resorption activity.

It is also possible that the formation of the ruffled border area is affected.

Izdebska et al. (2019) mentioned that viability of gliomaC6 cells decreased by 10 mM lidocaine, due to an unstable form of actin. This could also be the case in our cultures. Osteoclasts rearrange actin fibers to make a ruffled border area in which the protons are secreted to degrade the bone.

Lidocaine reduced the osteoclastic bone resorption but not osteoclast formation. This suggests that lidocaine is a good candidate to inhibit excessive bone degradation as in osteoporosis or periodontitis. Currently bisphosphonates are frequently used to inhibit excessive bone degradation in diseases such as osteoporosis. It is known that bisphosphonates cause osteoclast apoptosis and affect the coupling between osteoblasts and osteoclasts which besides less bone degradation also results in less bone formation (Jensen et al., 2021; Lacombe et al., 2013). This side effect is not present when treated with a low concentration of lidocaine. The osteoclasts are still present and the coupling between osteoblasts and osteoclasts still exists.

In conclusion, the combination of all results showed that lidocaine can reduce osteoclast activity. Long exposure to a high concentration of lidocaine may cause cell death of osteoclast precursors. When a lower concentration was used no differences in osteoclast number were observed however, the expression of inflammatory markers remained low and, when cultured on bone the osteoclast activity was significantly reduced. More research is needed to confirm these results. It is important to culture the cells in their natural environment such as bone or bone substitute. In the experiment on bone described here only PBMCs were cultured on bone. Since the lysosomes of fibroblast-like cells appear to be more sensitive to lidocaine, it is recommended to also use a co-culture on bone in future studies.

## CRediT authorship contribution statement

**Stan Tuijp:** Writing – original draft, Formal analysis, Data curation. **Marc Meijer:** Writing – original draft, Investigation, Data curation. **Ineke D.C. Jansen:** Writing – review & editing, Writing – original draft, Supervision, Project administration, Methodology, Investigation, Formal analysis, Conceptualization.

## Declaration of competing interest

Stan Tuyp, Marc Meijer and Ineke D.C. Jansen have no competing interests to declare.
Stan Tuyp: I have nothing to declare
Marc Meijer: I have nothing to declare
Ineke D.C. Jansen: I have nothing to declare.

## Data Availability

Data will be made available on request.
